# Massively parallel functional genomic assays in endocrinology: from promise to delivery

**DOI:** 10.1530/JME-25-0134

**Published:** 2026-06-05

**Authors:** Jingxiang Xu, Vahid Aslanzadeh, Grzegorz Kudla, Robert K Semple

**Affiliations:** ^1^ Institute for Neuroscience and Cardiovascular Research, University of Edinburgh, Edinburgh, UK; ^2^ Institute of Genetics and Cancer, University of Edinburgh, Edinburgh, UK

**Keywords:** MAVE, deep mutational scanning, hormone receptors, endocrine genetics, receptor signalling, saturation mutagenesis

## Abstract

The late 20th century saw major advances in mapping genes and mutations in endocrine disorders, driven by biochemical studies that identified defects endocrinologically and by family-based studies that mapped them genetically. In the 21st century, however, the world of genetics has been transformed by the mapping of the human genome and the development of next-generation sequencing technologies that allow genomes to be sequenced at population scale. This has led us to be swamped by a surfeit of information about genetic variation, with the so-called genetic ‘variants of unknown significance’ (VUS) greatly outnumbering pathogenic mutations in genes of clinical relevance. A revolution in functional genomics has since gathered pace, driven by the need to evaluate the function of rare genetic variants at scale. Recent advances in saturation mutagenesis enable the synthesis of libraries of thousands of gene variants. When coupled to increasingly creative use of a growing repertoire of downstream assays, this permits assessment of disease-relevant function of thousands of variants simultaneously. Such assays, widely known as multiplexed assays of variant effect (MAVEs), have major potential in endocrinology when applied to hormone receptors and their downstream signalling pathways. They help determine functional significance or otherwise of VUS, enable patient stratification for rare disease treatments, and yield new insights into structure–function relationships for target genes. We review the current state of development of MAVEs and their application to date to endocrine genetic disease, with particular reference to hormone receptors.

## Introduction

The molecular biology revolution ushered in an era of accelerating discovery in endocrine genetics in the late 20th century. In this phase of research, careful endocrine and biochemical investigation traditionally localised endocrine defects functionally before genetic studies, often conducted in rare families featuring a Mendelian pattern of disease. During this period, pathogenic variants were discovered in genes encoding peptide hormones or enzymes involved in hormone synthesis and in genes involved in the development or maintenance of endocrine glands or other hormone-secreting tissues. In such disorders, replacement of deficient hormones remained the mainstay of therapy.

Genetic discovery was particularly notable for its success in identifying loss-of-function variants in hormone receptors. By the turn of the century, pathogenic mutations had thus been described across multiple hormone receptor classes. These included nuclear hormone receptors (e.g. for thyroid hormones (1), androgens (2), oestrogen (3), mineralocorticoids (4), and vitamin D (5)), G-protein-coupled receptors (e.g. for TSH (6), ACTH (7, 8), and melanocortin neuropeptides (9, 10)), cytokine-type receptors (e.g. for growth hormone (11) or leptin (12)), and receptor tyrosine kinases (e.g. the insulin receptor (13, 14)). A smaller number of defects were also localised to genes involved in signal transduction downstream from receptors themselves and often shared by several receptors. Examples of these were defects in *GNAS* (15), encoding the Gsα G-protein subunit, and *PTEN* (16), a negative regulator of phosphoinositide 3-kinase (PI3K) signalling.

For all this success, endocrine genetic research was arduous and progress still relatively slow. This was due in large part to lack of a reference map of the human genome and to reliance still on the Nobel-prize-winning but onerous DNA sequencing methodology developed by Frederick Sanger in 1977 (17). At the start of the 21st century, however, dramatic technical advances removed these bottlenecks. First, the success of the multinational Human Genome Project provided the missing map of the human genome (18), and second, a raft of ‘next-generation’ sequencing (NGS) technologies was developed permitting massively parallel sequencing on a hitherto unimaginable scale (19). NGS was first used successfully to identify unsuspected pathogenic genetic variants in 2009 (20), and the pace of discovery has since been breathtaking as technology has been refined and rolled out at scale. Exome-wide and/or whole genome sequencing are now applied not just in people with a high pretest likelihood of genetic disease but also in those for whom clinical suspicion is much lower, and increasingly also in large general population samples.

**Table:** 

**GLOSSARY OF KEY TERMS**
**NGS:** Next-generation sequencing
**VUS**: Variant of unknown significance
**VEP**: Variant effect predictor; a computational tool used to predict functional consequences of gene variants
**MAVE**: Multiplexed assay of variant effects
**DMS**: Deep mutational scanning
**Saturation mutagenesis**: Introduction of all possible nucleotide substitutions within a genomic region of interest; for coding genes often all possible amino acid substitutions
**ClinVar**: A public database that aggregates and archives genomic variants and their reported relationships to disease (21) (https://www.ncbi.nlm.nih.gov/clinvar/intro/)
**GnomAD**: The genome aggregation database, a resource that compiles and harmonises human genetic variation data from large-scale sequencing projects to provide population allele frequencies (22) (https://gnomad.broadinstitute.org/about)
**Nonsense variant**: Nucleotide substitution that changes an amino acid-encoding codon to a stop codon, resulting in truncated or absent protein product
**Missense variant:** Nucleotide substitution that changes an amino acid-encoding codon to encode a different amino acid, leading to an altered protein sequence
**Synonymous variant:** Nucleotide substitution that does not change the amino acid encoded by the codon
**Dominant negative mutation:** A mutation that yields a protein which not only has impaired function but also interferes with the function of co-expressed wild-type protein. This means that a heterozygous mutation may produce >50% loss of function
**Haploinsufficient**: A term applied to genes where 50% loss of function (for example, heterozygosity for an early stop mutation) is sufficient to produce a disease phenotype
**ACMG/AMP criteria**: A set of criteria developed by the American College of Medical Genetics and Genomics (ACMG) and the Association for Molecular Pathology (AMP) to assess probability of pathogenicity of a gene variant, drawing on multiple, weighted evidence types

**Figure 1:**
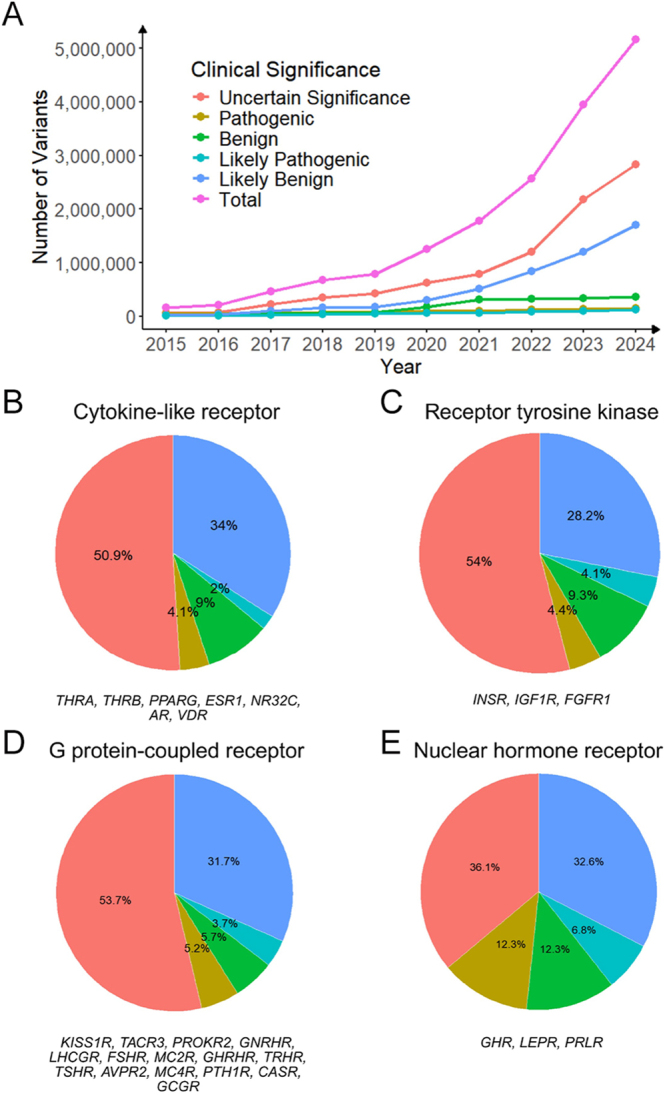
Diagnostic challenge of variants of unknown significance (VUS) (A). Cumulative numbers of rare variants curated into different diagnostic/functional categories in the ClinVar database since 2015, illustrating that the exponential rise in detected variants is largely accounted for by VUS. (B, C, D, E) Breakdowns of variant types by hormone receptor class for groups of receptors with well-established roles in monogenic endocrine disease. Genes included are shown below each pie chart (ClinVar accessed 12 April 2025).

As the cost of NGS has dropped, and as its throughput and precision have increased, the effect of these saltatory technical advances has been to increase the rate of pathogenic mutation discovery explosively, as hoped and expected. However, they have also created new barriers to realising the full potential of genomics in endocrinology. In particular, although the number of *bona fide* disease-causing gene variants described has indeed increased dramatically, so has documentation of background genetic variation in the human genome (21, 22). This has created a major ‘signal-to-noise’ problem in discerning convincing pathogenic gene variants among a sea of incidental variants, for which there is no clear understanding of biological or clinical significance. Such variation is universal, with every person sequenced estimated to harbour 20,000 rare coding variants (23). Most gene variants identified in diagnostic sequencing are thus labelled as ‘variants of uncertain significance’ (VUS). Documented VUS have rapidly outstripped pathogenic variants in widely used clinical genomic databases such as ClinVar (Fig. 1) and now pose a major challenge in genetic diagnosis (24).

Computational algorithms are one approach to managing this surfeit of genomic data. These are now used routinely, usually in aggregate, in diagnostic pipelines to support pathogenicity (25, 26). Many computational variant effect predictors (VEPs) have been described, with variable diagnostic performance. New VEPs continue rapidly to be developed, with many, like AlphaMissense, now utilising deep learning techniques that offer significant improvements over earlier heuristic-based – that is, involving computational ‘trial and error’ (27, 28) – approaches. Despite these rapid advances, current VEP algorithms still have technical limitations, and moreover, according to current guidelines VEP-derived functional predictions are used only as supporting evidence for pathogenicity (25). Problems with VEPs include overfitting towards existing clinical annotations (29), biased performance in populations underrepresented in sequencing data (30), and variable predictive performance across genes due to inadequate calibration of scores (31).

A more nuanced limitation of VEPs is that they usually take gene/protein ‘function’ to be unidimensional. This takes no account of the possibility that mutation of multifunctional proteins may impair only one, or a subset, of all the protein’s normal functions, or where different functions are affected to different degrees. This is clinically important as such genes often exhibit distinct genotype–phenotype associations, with different mutations sometimes yielding diseases affecting different developmental or physiological processes. One striking example is *PIK3R1*, which encodes a regulatory subunit of phosphoinositide 3-kinase (PI3K), a crucial player in signalling from many types of hormone receptor: early nonsense *PIK3R1* mutations produce immunodeficiency and enhance insulin sensitivity, while nonsense mutations late in the gene cause severe insulin resistance and developmental defects. Furthermore, missense mutations produce immunodeficiency, developmental defects, severe insulin resistance, or vascular overgrowth, all depending on the variant and domain of the protein affected (32). This illustrates how mutations in the same gene can result in a wide spectrum of phenotypic outcomes.

Finally, even where there *is* only a single gene function relevant to disease, VEPs fail at present to predict the molecular mechanism underpinning predicted loss-of-function. This limits attempts to develop precision therapies for rare endocrine diseases. In the case of hormone receptors, an expressed receptor that fails to respond to its endogenous ligand may still respond to an atypical ligand. However, assessing such novel ligands would be futile in cases where no mature receptor protein is expressed. On the other hand, in this case, attempts to boost protein expression, for example, using small molecule chaperones may be worthy of testing (Table 1).

**Table 1:** Translational implications of different disease mechanisms associated with hormone receptors.

Overall effect of mutation on receptor function*	Molecular mechanism of impaired function	Rational translational strategies to mitigate impaired receptor signalling for therapeutic benefit				
		Atypical agonist therapy	Chaperone^†^	Readthrough-inducing agent^‡^	Nucleic acid therapy to reduce mutant allele expression	Engaging parallel compensatory pathways
Loss of function	Impaired protein expression	No	For missense variants	For nonsense variants	No	Yes
Loss of function	Impaired hormone binding by expressed mutant receptor	Yes	Only if also reduced expression (missense)	Only if also reduced expression (nonsense)	Yes, if: A. Dominant negative effect B. Little/no haploinsufficiency	Yes
Loss of function	Impaired signalling by expressed mutant receptor that binds hormone	Yes	Only if also reduced expression (missense)	Only if also reduced expression (nonsense)	Yes, if: A. Dominant negative effect B. Little/no haploinsufficiency	Yes

* For example, as yielded by a variant effect predictor (VEP).

^†^ Chaperones are small molecules that aid normal protein folding.

^‡^ Readthrough-inducing agents are small molecules that increase the likelihood of protein synthesis occurring through a premature stop codon.

**Table 2:** Attributes of organisms and cells commonly used for MAVEs.

Organism/cells		Key attributes
Yeasts		High ease of genetic manipulation Flexibility in design of survival assays (e.g. nutritional, dependent on transcription factor binding of promoter driving antibiotic resistance gene expression; split protein complementation assays) Maintainable in haploid life cycle More variable relevance to human disease
Mammalian cells	Diploid	Many cell lines are robust to the extensive manipulation required, often including single cell expansion Two alleles of autosomal genes complicates MAVE design and interpretation if both are expressed Endogenous alleles are commonly knocked out or knocked down to give clean background for MAVE
	Haploid	No compensation by co-expressed WT allele Amenable to *in situ* editing and survival assays
	Induced pluripotent stem cells	Potential to model a wide range of developmental disorders High cost of culture *In situ* editing and exogenous library expression often more challenging than in immortalised cell lines, in part due to promoter silencing Long differentiation protocols and developmental bottlenecks may attenuate library diversity

It follows from these observations that there is a pressing need to characterise the functional consequences of as many VUS as possible experimentally, to guide both research and clinical decision-making (33). Investigating each new variant discovered individually is resource-intensive, and not feasible, however. This new bottleneck has supplanted pathogenic mutation discovery as the rate-determining step in clinical diagnosis, downstream cascade testing and translational research in many cases of rare monogenic diseases. It is this gap that the rapidly developing field of multiplexed assays of variant effect (MAVEs) seeks to fill.

## Multiplexed assays of variant effect

MAVEs provide a functional readout for large numbers of variants in genes of interest simultaneously, sometimes referred to as fitness landscapes (34), often on a scale of many thousands of variants (35, 36). Nomenclature around this experimental approach is not yet settled, and the process of undertaking MAVEs is also commonly described as deep mutational scanning (DMS), or sometimes residuomics (37).

The possibility of massively parallel functional assays of libraries of genomic variants had been discussed for many years prior to the current widespread enthusiasm, but it has taken recent major developments in synthetic biology and genome editing for them to fall within reach of large numbers of laboratories. It is now possible to quickly and accurately generate extremely large libraries of permuted DNA sequence variants, most commonly for protein-coding genes. This can be achieved either *in situ* at the normal genomic location or in plasmids containing complementary DNA (cDNA). Where an assay can be designed that gives a disease-relevant functional readout at the desired scale, the effect on protein products of each gene variant can then be determined.

Rigorous functional studies are considered strong evidence (PS3/BS3) by the widely used American College of Medical Genetics and Genomics/Association for Molecular Pathology (ACMG/AMP) genetic diagnostic guidelines. In the context of a Bayesian decision-making framework they can effectively play a deterministic role in classifying variants (25, 38, 39), thereby potentially making major inroads into the ‘VUS problem’. The ACMG/AMP guidelines continue to evolve, and at the time of writing revision is underway, with an update to version 4 anticipated shortly. ‘Mainstreaming’ of MAVEs in diagnostic pipelines is also facilitated by community resources managed under the umbrella of an Atlas of Variant Effects alliance (40). These include MaveDB (41), a repository of completed MAVE studies and associated data, MaveRegistry (42), which seeks to facilitate co-ordinating, prioritisation and deduplication of ongoing studies, and recently MaveMD, an extension of MaveDB that transforms MAVE functional scores into calibrated ACMG-compatible evidence (105). These have made the initial effort to promote collaboration and translational application of MAVE studies, centralising and standardising functional variant data to enable systematic access to experimentally measured variant effects. Nonetheless, how to standardise design and reporting of MAVEs, how best to use MAVE-derived data to support clinical decision making, and how to build user confidence in the rigour of MAVE-based functional findings are subjects of continuing deliberation (39).

Importantly, MAVEs should not be regarded simply as competition for VEPs, but rather as complementary to them, with huge potential for synergy between experimental and computational approaches. This is illustrated by the finding that results from VEPs and MAVEs, while showing broadly similar, and usually high, overall predictive performances, actually show only modest correlation: MAVE-VEP correlation coefficients (Spearman ρ) were around 0.5–0.6 for a raft of top-ranking VEPs in one recent study (43). Importantly, MAVEs generate large-scale, experimental datasets with ‘low circularity’ (i.e. with minimal or no dependence on the data sources commonly used by VEPs) benchmark for VEP validation (29). The true scale of potential synergy is only beginning to be realised, however (44).

## Strategies for mutational library generation

**Figure 2:**
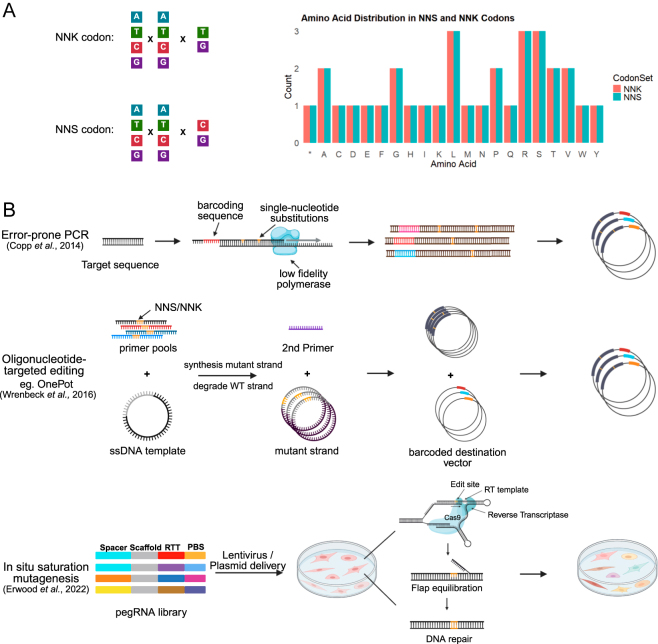
Commonly used strategies for DNA library creation for MAVEs (A). Distribution of NNS- and NNK-encoded amino acids. (B) Example methods for each category of strategies used to generate DNA variant libraries. *: Stop codon; WT: wild type; RT: reverse transcription; RTT: reverse transcription template; PBS: primer-binding site. “Commonly Used Strategies for DNA Library Creation for MAVEs” created in BioRender. Xu, J. (https://BioRender.com/fdqjod1), licensed under CC BY 4.0.

The generation of a variant library is the first step in any MAVE, often involving saturation mutagenesis to introduce nucleotide changes in a target gene or gene region, resulting in a comprehensive library of variant proteins derived from the open reading frame targeted. Mutagenesis can be achieved using different approaches, each with distinct advantages and limitations. These methodologies collectively offer a diverse set of tools for constructing variant libraries, enabling precise and high-throughput functional genomic studies (Fig. 2).

An important group of methods are based on polymerase chain reaction (PCR). Error-prone PCR is a common method, relying on the imperfect fidelity of engineered DNA polymerases. However, this tends to produce single-nucleotide substitutions, limiting its utility as this cannot produce the full repertoire of amino acid substitutions at each codon (45, 46). Alternatively, oligonucleotide-targeted editing offers greater flexibility. In this approach, desired mutations are incorporated in specially synthesised oligonucleotide primer pools, which are then used for PCR employing high fidelity polymerases. Specifically, for codon mutagenesis, oligonucleotides are designed with one degenerate codon (e.g. NNS, NNK) in each pool, and one pool for every codon to be mutated. NNS and NNK codons encode all possible amino acid changes at the target codon while minimising stop codons. Reported examples of such mutagenesis methods include POPCode (47), PFunkel (48), OnePot (49), PALS (50), and inverse PCR (51), all of which permit precise and massively parallel mutagenesis (Fig. 2).

Customised site saturation variant libraries can also be fully synthesised to order (52). These libraries eliminate codon bias, allow equal representation of each amino acid substitution, and substantially reduce the time required to optimise and implement saturation mutagenesis experiments. Such libraries are now favoured by many experienced MAVE laboratories (53); however, limited target length and cost considerations still impede global adoption in the field (52).

A final important group of approaches employ CRISPR-related technologies to effect *in situ* saturation mutagenesis at the endogenous locus. Such saturation genome editing (SGE) is the most adopted *in situ* editing method, involving co-delivery of a CRISPR–Cas9/sgRNA complex and a mutagenising repair template library. Cas9 introduces a targeted double-strand break, which is repaired by homology-directed repair using the donor templates, resulting in the incorporation of a single variant at the endogenous genomic locus (54, 55). Unlike SGE, CRISPR prime editing enables *in situ* mutagenesis without requiring homologous donor templates. In saturation prime editing, cells are delivered a prime editor, comprising a Cas9 nickase fused to a reverse transcriptase, together with a library of prime editing guide RNAs (pegRNAs) encoding the desired mutations. Each pegRNA directs editing to the target locus and provides the reverse-transcription template needed to install the programmed variant at the endogenous site (56, 57) (Fig. 2B). Endogenous genome-editing approaches preserve the native genomic context, including introns and regulatory elements. Consequently, these approaches enable the detection of splice-disrupting synonymous and intronic variants, which cannot be readily assessed using cDNA-based library generation methods in MAVE studies (58).

## General design considerations for multiplexed functional assays

Development of an assay that i) reports on a disease-relevant function of the gene product of interest and ii) can be used at enormous scale is crucial to MAVE design. For each position in a protein, there are 20 possible amino acids plus a potential stop codon, so for a 100 amino acid protein, there are 2,000 potential variants, which increases when insertions and deletions are included in the mutational library. For a 1,000 amino acid protein, there would thus be 20,000 protein variants, as well as a large number of wild-type protein copies derived from a sequence with synonymous variants. Moreover, to reduce sampling noise, multiple cells expressing each variant, ideally encompassing multiple clones, should be measured in a single experiment. This means that millions of cells may be required per experiment for larger genes. Finally, sequencing depth must be tailored to variant abundance. Some protocols further reduce error by employing barcoding strategies (59), where variants are tagged with two or more unique barcodes, enabling noise reduction through averaged fitness scores across technical replicates.

The first key consideration in assay design is thus choosing a model system in which the desired scale of the library can be accommodated and expressed. The second key design consideration is whether or not the functional readout of the chosen downstream assay is relevant to the clinical condition or phenotype being studied (38). These will be considered briefly in turn.

### Choice of cell/organism type

MAVEs may, in principle, be implemented in a wide range of model systems, ranging from acellular systems through bacteria, plants and lower eukaryotes to mammals *in vivo *(Table 2). The well-established approach of (bacterio)phage display, developed in the 1980s, can be regarded as an *in vitro* MAVE prototype (60, 61). In this approach, expression libraries of variants in a gene of interest fused to a phage gene are used to direct expression of the fusion protein product on the exterior of the phage. This lends itself to cycles of ‘panning’ against immobilised substrate, interspersed with amplification of selected phages in a bacterial host. This form of directed evolution has been powerfully applied to developing and maturing monoclonal antibodies and was recognised as part of the 2018 Nobel Prize for Chemistry (62). More recently, an acellular cDNA display proteolysis system has been used to measure the thermodynamic folding stability of nearly a million protein domains in a single experiment (63).

MAVEs have also been used extensively in bacterial models, often to interrogate mechanisms of antibiotic resistance (64); however, it is eukaryotic cellular models that have the greatest relevance to endocrine scientists. The simplest widely used eukaryotic models are yeasts, which offer a simple, cost-effective, and powerful system for high-throughput studies. Yeast surface display, in particular, has been used to screen thousands of protein variants for binding affinity and function (65, 66). Yeast models have also been used to screen human disease genes in complementation assays, where the human gene functionally replaces a homologous yeast gene essential for growth (47), as well as protein–protein and protein–DNA interactions (67, 68). The use of yeast for enzymatic protein functional assays relevant to endocrinologists was exemplified by study of the phosphatase encoded by PTEN, which antagonises PI3K signalling, and which is mutated in the PTEN Hamartoma Tumour Spectrum (69).

Although less experimentally tractable than bacteriophages, bacteria, and yeasts, mammalian cells provide a closer approximation to human tissue, and have been used most widely for endocrinology-relevant MAVEs. The complexity and higher costs involved in working with mammalian cells’ constrain scalability, and strategies both for cellular library creation and downstream assay need to be developed carefully, mindful of the required variant library size. Considerations such as ease of transfection and/or *in situ* gene editing, robustness to extensive manipulation, which may include clonal expansion, and endogenous expression of the gene of interest are key. Where *in situ* editing is used, endogenous expression of the target gene is a prerequisite for an effective MAVE. In contrast, where the gene of interest is expressed transgenically, endogenous expression can be a major confounder, usually dealt with by endogenous gene knockout or silencing.

Many mammalian cell lines have been used in MAVEs. A selection of these is discussed later in the context of endocrine genes; however, a few of cell lines are worthy of particular note. Kidney-derived HEK293 cells are the ‘workhorse’ mammalian cell model for MAVE given their ease of culture and transfection, rapid growth, and ready adaptation to pooled, high-throughput assays – all while providing a human cellular context that better captures protein folding, post-translational modification and intracellular signalling than bacteria or yeast. HEK293 cells have moreover been modified to optimise variant library integration, rendering them particularly useful for generic functional assays such as VAMP-seq, which determines variant effects on protein abundance (70, 71).

Also widely used in MAVEs are spontaneously haploid mammalian cells such as the myeloid lineage Hap1 line (36). Use of such lines eliminates the confounding effects of a second allele, and such lines are particularly amenable to genome editing approaches. MAVEs in haploid cells are also informed by well-curated lists of genes which are essential to survival of such cells (72, 73). Together, these factors have led to MAVEs in Hap1 cells being semi-automated for large-scale deployment in functional genomics (74). However, few, if any, *bona fide* hormone receptors are essential for the survival of Hap1 cells (72, 73).

A third type of mammalian cells with high translational potential are induced pluripotent stem cells (iPSCs). iPSCs, with their inherent capacity for directed differentiation into a large number of mature cell types, excite particular interest because of the vast scope they offer for MAVEs in different lineage contexts. iPSCs have already been employed in large-scale CRISPR screens, suggesting that similar strategies could be adapted and integrated into future MAVE experimental designs (75, 76, 77). The well-established examples of iPSC-derived cells of potential endocrine interest include pancreatic beta cells (78), adipocytes (79), thyroid follicular cells (80), steroidogenic adrenal cortex cells (81), and liver cells (82), while iPSC-derived organoids have also been described for various hormone-secreting organs (e.g. thyroid (83), pancreatic islet (84), pituitary (85), adrenal (86), and target tissues (e.g. liver (82), adipose tissue (79)). Despite the huge potential of MAVEs based on iPSCs, significant barriers currently exist to their wide scale development. These include the high cost of iPSC culture, the challenge of efficient gene editing of iPSCs, the silencing of many promoters in iPSCs, especially in the context of viral sequences, although recent progress has been made towards overcoming these problems (87). A further challenge is that iPSC differentiation protocols are frequently long and labour-intensive, often with bottlenecks that attenuate library diversity. Nevertheless, these challenges are being circumvented, and the power and versatility of iPSCs in MAVEs are now beginning to be revealed (88). Rapid further progress is anticipated.

### Choice of functional assay

Creating a cellular library harbouring the full diversity of variants of interest, including sufficient replicates and controls, is only the first step in MAVE design. The likely statistical robustness of the MAVE, and thus the requisite number of replicates and controls, can only fully be modelled once the performance characteristics and disease relevance of the downstream assay are understood. Attempts to develop standards, and a statistical framework, for assessment of the diagnostic performance of functional assays lagged somewhat behind efforts to curate and assess gene variants, but a rigorous approach to this has been proposed (38).

Core requirements of the functional assay chosen are that i) it must allow for linking of the phenotypic outcome for each cell to the mutation harboured by that cell, ii) it must be relevant to the disease or *in vivo* process of interest, and iii) it must offer sufficient dynamic range to discriminate signal from noise and ideally different degrees of loss of function.

Linking outcomes to mutations is generally achieved through next-generation sequencing approaches. In principle, these could involve sequencing of the whole gene of interest in all cells at the end of the assay. This is the most common approach in MAVEs based on *in situ* editing, where targeting exon by exon is employed, and the exon concerned can be sequenced. For many MAVEs based on transgenic expression sequencing, the whole open reading frame is precluded by cost, especially for large genes. In such MAVEs, the incorporation of scrambled ‘barcode’ DNA sequences in *cis* with each point mutation is thus commonly employed in library creation. Such barcodes, usually placed adjacent to coding sequence, and sometimes incorporated in transcribed mRNA, have immense diversity – even a 20 oligonucleotide barcode, with any of the 4 nucleotides at each position, has more than 1 trillion (10^12^) permutations. In the process of library validation, long-read DNA sequencing can be used to ‘phase’ barcodes and variants – that is, to create an index listing the barcode(s) associated with every variant. Once this mapping has been established, variant abundance in the functional assay can be quantified by sequencing only the short barcode region, rather than the full variant sequence (89). Various free tools integrating the full downstream processing pipeline including barcode-variant phasing, counting barcode abundance, collapsing of multiple barcodes per variant and calculating variant score are now available (89).

**Figure 3:**
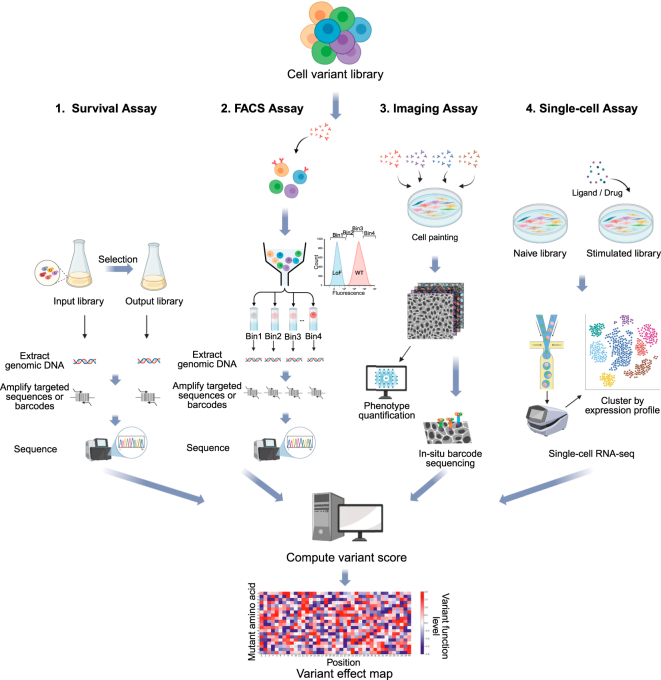
Types of functional assays widely adapted for MAVEs. Typical workflows for different types of MAVEs. In survival-based assays, the output libraries are often collected at many different time points, permitting some assessment of the severity of variant effects. In FACS-based assays, cells are often collected in four bins as FACS sorters commonly support no more than four-way sorting. More bins could be used if biologically meaningful and if hardware supports this. “Types of Functional Assay Widely Adapted for MAVEs” created in BioRender. Xu, J. (https://BioRender.com/v5bd4aj), licensed under CC BY 4.0.

The assay employed and its relevance to disease depend exquisitely on the gene and disease concerned. However, the exacting requirement to study vast numbers of cells – often numbering many millions – has practically constrained assays to several broad types (Fig. 3).

#### Survival assays

Cellular survival assays, whether in yeast or in mammalian cells, can provide a powerful platform for identifying damaging variants in disease genes. These assays first require identification of a cell type and associated culture conditions that render cell survival or replication dependent on the intact function of the gene of interest. This can directly exploit the native protein function in some cases, or instead can utilise approaches such as abundance protein fragment complementation assay. In these assays a protein essential for cell survival is split in two, with one fragment fused to the gene being subjected to the MAVE, when the fused protein product is expressed and stable, the fragments of the divided protein can bind, restoring function of the essential gene. This approach, although more contrived and indirect, is powerful and generalisable and has been used at scale (90).

In many survival-based MAVEs, yeast in their haploid state or near-haploid cell lines, such as Hap1, are a particular asset, as diploid cells require biallelic knockout of the gene of interest to demonstrate its essentiality. Where this condition is met, the ability of cells harbouring gene variants to persist in a population under this selective pressure indicates that function is preserved. By sequencing the variant library of cells before and after selection, damaging variants can be identified if they disappear or become progressively less represented under these conditions of directed evolution (68). In their simplest implementation, using a single time point, survival assays have a tendency to dichotomise outcomes; however, the use of time-course survival assays, or varying doses of cellular stressor, can discriminate mildly deleterious mutations which cause gradual fitness decline from those with strong loss-of-function effects (36).

#### Fluorescence-activated cell sorting (FACS)

FACS-based assays enable functional classification of variant libraries by sorting cells into different bins based on fluorescence intensity (91). For this to be meaningful, fluorescence must be entrained to protein expression and/or function, leading to a clear separation of cells null for the gene of interest and those expressing wild-type protein on sorting. Common strategies involve fusion of the gene of interest to a gene encoding a fluorescent protein, with fluorescence used as a surrogate for expression of the target protein, in the assay subtype known as VAMP-seq (variant abundance by massively parallel sequencing), which has been optimised and validated across multiple targets (70). Another strategy involves the use of a fluorophore-labelled ligand or antibody that binds specifically to the relevant gene product itself (e.g. (59)) or to a downstream upregulated protein (e.g. (84)). This works best for cell surface proteins, but can sometimes be used with fixation and permeabilisation for intracellular proteins (e.g. (59)). If a variant disrupts protein function or expression, it will reduce or eliminate binding of fluorophore, redistributing cells away from the wild-type towards null control cell bins. Intermediate loss-of-function variants will distribute between null and wild-type bins, which can be captured by sampling cells also from intermediate bins. By amplifying barcodes and sequencing them from each bin, then using the predetermined barcode-variant index to determine the distribution of each variant across bins, a functional score can be computed for each variant (e.g. (59, 92, 93)).

#### Imaging-based assays

In recent years, next-generation sequencing approaches have been adapted to enable *in situ* sequencing of DNA barcodes tagging imaged cells (e.g. (94, 95)). Although originally developed to allow imaging-based, pooled CRISPR screens, such approaches also offer powerful possibilities for multidimensional MAVEs. High content imaging of cell populations harbouring mutation libraries can either be directed at imaging cell phenotypes of specific interest and disease relevance, or can be used more agnostically with multiple organelle stains to discriminate different patterns of abnormality. This may, for example, involve simultaneous examination of nuclear shape, mitochondrial organisation, cytoskeletal architecture, membrane integrity, or other cellular features arising from different mutations. Such multiparametric high-content cell painting (96) opens the possibility to quantify multiple cellular phenotypes simultaneously, potentially adding value in the study of genes with more complex genotype–phenotype relationships. Such imaging approaches as MAVE readouts has recently been applied successfully to *LMNA* and *PTEN*, both genes with complicated genotype–phenotype relationships and endocrine disease associations (97). By sequencing barcodes *in situ*, registration of barcodes to images is ensured. Such MAVEs do not yet approach the throughput of survival or FACS-based assays, but technology is still rapidly developing.

#### Single-cell transcriptomics

Single-cell RNA sequencing (scRNA-seq) simultaneously determines expression of thousands of genes at the individual cell level (98). When library design ensures that barcodes tagging variants are well expressed and detectable by scRNA-seq, this offers another powerful high-dimensional route to discriminate many different disease-related phenotypes. This approach is particularly powerful for studying a gene which has significant effects on gene expression, for example ligand-activated transcription factors such as nuclear hormone receptors, where selective modulation of function by different ligands is well documented. To date, however, some of the best examples have studied oncogenes or tumour suppressor genes, such as p53 (99).

## Data analysis, standards and reproducibility

Assigning a score to each variant in a MAVE is based on their enrichment, or enrichment of their tagging barcode, in the cell population after selection, whether this be in a survival, FACS or other assay paradigm. At least seven different MAVE data analysis tools have been released (89); some are able to analyse experiments with multiple time points or conditions and some are limited to analysing single pre-selected and post-selected cell populations. Examples include TileSeqMave v1.0 (47), commonly used in MAVEs employing a direct/tile sequence approach, and Enrich2 (100), widely adopted for MAVEs utilising barcode sequencing (89).

The rapidly expanding MAVE field has tended to prioritise novel MAVE designs and/or MAVEs of hitherto unstudied genes to date. This rapid staking out of the technical frontier has left rigorous addressing of MAVE reproducibility somewhat behind. Individual studies have taken a variety of approaches to statistical analysis of within-study variability, while few examples – and none for hormone receptors – exist of genes studied in different model systems or laboratories. Two independent MAVEs of Parkin using different cellular systems and assay designs showed strong agreement (*R* = 0.77) and high diagnostic performance (ROC AUC 0.92 and 0.94), despite differences in library design, functional readout, and model system (101, 102). In contrast, abundance-based assays such as VAMP-seq showed lower classification performance (ROC AUC ∼0.70–0.78), suggesting that assays measuring protein function directly generally provide larger effect sizes and improved variant discrimination compared to stability or abundance assay (70).

A key step taken by the community towards meeting the needs for cross-study standardisation and a data repository has been the creation of the publicly accessible MaveDB database (41). This follows strict data and documentation standards, mandating HGVS nomenclature for variant representation and embodying FAIR (findable, accessible, interoperable, and reusable) principles for data sharing, and compliance with GA4GH (Global Alliance for Genomics and Health) frameworks. These standards ensure capture of essential details of experimental design to ensure reproducibility and integration with other resources such as ClinVar (24) and gnomAD (103). The inclusion of application programming interfaces and visualisation tools further simplifies data analysis, making MaveDB a valuable resource for the genomics community (41). MaveDB has recently been developed further for translational purposes with the release of a clinical layer of the database – MaveMD – which transforms MAVE functional scores into calibrated ACMG-compatible evidence that clinicians can use to support variant classification (104). Such efforts to standardise data analysis and reporting are major steps towards the realisation of the clinical diagnostic potential of MAVEs; however, there remains a pressing need for more replication studies of the same genes across models and laboratories.

Other translational barriers related to performance evaluation also remain. The size of mutational truthsets used for validation and testing of the discriminatory performance of any MAVE has emerged as a key issue. Modelling based on ACMG/AMP criteria, as applied to cancer-related variants, has shown that truthsets of benign variants are much more limiting than those for convincing pathogenic variants (105). Relatively few such benign variants have been functionally validated, while curation of pathogenicity in ClinVar is not based on rigorous functional data. The need to develop high-confidence, benign truthsets has thus been identified as a key need for the community. A pragmatic solution to the problem has been proposed based on proactively generating benign variant lists based on factors such as population frequencies, disease rarity, and ACMG/AMP criteria (105).

## Application of MAVEs to hormone receptors

**Figure 4:**
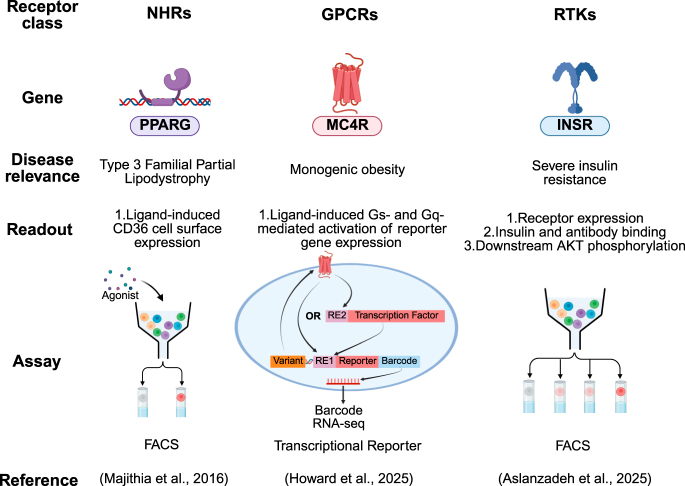
Examples of endocrine receptor genes subjected to MAVE readouts for Gs and Gq signalling were obtained using different reporter systems: Gs signalling directly activated reporter gene expression, whereas Gq signalling required a two-step reporter system to amplify the signal. FACS: fluorescence-activated cell sorting; GPCRs: G-protein-coupled receptors; NHR: nuclear hormone receptors; RE: responsive element; RTKs: receptor tyrosine kinases. “Examples of Endocrine Receptor Genes Subjected to MAVEs” created in BioRender. Xu, J. (https://BioRender.com/sqrgdwa), licensed under CC BY 4.0.

The application of MAVEs to endocrine disorders is in its infancy, yet the potential is immense. As well as the general aim of mitigating the VUS problem in diagnostic sequencing of endocrine disease genes, MAVEs of hormone receptors present particularly appealing translational opportunities. Many of these arise because of the high tractability of targeting receptors with ligands that may be endogenous, modified versions of endogenous ligands, or wholly synthetic. This gives great scope to pan different agonists against comprehensive mutational libraries of receptors of interest, serving to identify potential beneficial actions of some ligands to activate specific receptor mutations. This may not only be informative about receptor structure–function relationships but may also stratify all possible mutations *a priori* in terms of different ligand responsiveness, obviating the need to study every mutation functionally as it is identified. This helps to tackle a rate-determining step in rare disease translational research. Several examples of MAVEs applied to receptors of interest to endocrinologists, and representing different receptor classes, have been published to date (Fig. 4). Although different performance metrics were reported for each receptor MAVE, illustrating the evolving approaches taken in the field, in all cases discriminatory performance was high, with areas under ROC curves for identifying pathogenic mutations generally in excess of 0.90 (43, 59).

### Nuclear hormone receptors (NHRs)

NHRs are ligand-activated transcription factors that regulate gene expression in response to lipophilic hormones including sex steroids, mineralocorticoids, glucocorticoids and thyroid hormone. No receptors for such canonical hormones have yet been subjected to a MAVE; however, another important member of the nuclear hormone receptor family that is of endocrine relevance, namely PPARγ, has. PPARγ plays a crucial role in metabolic homeostasis and is the master transcriptional regulator of adipocyte differentiation. In keeping with this, loss-of-function PPARG mutations give rise to familial partial lipodystrophy (105). Whether or not a specific single ligand exists, or whether instead PPARG serves as a sensor of flux of a range of lipid species remains to be fully determined. PPARG was subjected to one of the earliest MAVEs applied to an endocrine disease gene (106). This MAVE used a tiled approach to library generation, with downstream assay in *PPARG* knockout monocytes. This assay used FACS to assess upregulation of CD36, a PPARG-responsive cell membrane lipid transporter and scavenger receptor, in response to two concentrations of two different ligands. MAVE data were further utilised to train a supervised machine learning classifier of the pathogenicity of PPARγ variants. This has been used subsequently to support clinical genetic diagnosis of familial partial lipodystrophy type 3.

### G-protein coupled receptors (GPCRs)

Membrane-associated GPCRs form the largest protein family. They transduce not only diverse extracellular cues, including photons, volatile odorants, inorganic ions, chemokines, and pH, but also, crucially for endocrinology, an important array of aminergic (e.g. dopamine, serotonin) and peptide (e.g. adrenocorticotropic hormone, thyroid-stimulating hormone, glucagon, the melanocyte-stimulating hormone family) hormones and neuropeptides/neurotransmitters, and small molecules of metabolic relevance (107).

Two GPCRs of endocrine interest have been subjected to MAVEs to date. The first of these, the beta-2 adrenergic receptor (β_2_AR), which mediates some actions of epinephrine in particular, mainly acts via Gα_s_ G-proteins to increase intracellular cAMP and thus protein kinase A (PKA) activity. This well-defined action was exploited to develop a cAMP-responsive transcriptional reporter system expressing a variant-specific barcoded reporter gene, which could be quantified as a readout of ligand-stimulated downstream β_2_AR signalling (108). This study delineated a novel and conserved structural latch important for receptor signalling, while also suggesting a generalisable platform for MAVEs of Gα_s_-coupled GPCRs.

This approach was further developed to study another important ‘endocrine’ GPCR, the melanocortin-4 receptor (MC4R) (109). MC4R is the most commonly mutated gene in monogenic obesity (110). MC4R signalling involves multiple G-protein-mediated pathways, including Gsα/cAMP and Gαq/PLC signalling, and different patterns of signalling bias among different pathways are associated with distinct phenotypes and treatment responses (111, 112). This study refined the cAMP reporter construct to improve signal-to-noise ratio and also developed a two-step reporter of Gαq signalling. The initial unacceptably weak signal from an NFATc-driven promoter was amplified by being used to induce a strongly activating artificial Gal4-Vpr chimeric construct. This, in turn, activated expression of a barcoded reporter distinct from that used to read out cAMP. Using this approach, the response of MC4R variants to two types of drugs with both Gs and Gq-mediated effects was mapped (109). Mutations skewing MC4R signalling and mutations selectively disrupting agonist binding were both identified, informing on mechanisms of ligand binding and signal transduction. These will inform drug design and aid patient stratification for therapy with those new drugs.

### Receptor tyrosine kinases (RTKs)

RTKs, most prominently represented in endocrinology by insulin and IGF1 receptors, engage signalling pathways, most prominently including the phosphoinositide 3-kinase (PI3K) and RAS/MEK/ERK pathways. RTKs are also critically important in cancer pathogenesis, where activating mutations in many growth factor-activated RTKs promote tumour growth. Nevertheless, MAVEs for RTKs are less well developed than for GPCRs, until recently focusing on mechanisms of receptor activation and resistance to a panel of RTK inhibitors, exemplified by studies of the MET receptor for hepatocyte growth factor (113). Rather than using reporter assays, these studies exploited the dependence of the Ba/F3 cell line on pathologically activated MET signalling for survival, supplanting the dependence of wild-type cells on IL-3.

More recently, the insulin receptor (INSR) has also been subjected to a multidimensional MAVE; using a barcoded library of extracellular variants expressed in mouse embryo fibroblasts in which Igf1r was knocked out and the endogenous Insr was knocked down (59). This study used FACS in conjunction with binding of fluorophore-labelled insulin, anti-INSR monoclonal Abs, or an antibody against the key signalling intermediate pAkt to assay cell surface expression, insulin binding, and, critically, maximal insulin or antibody-induced signalling (59). As well as yielding novel findings related to INSR structure–function relationships, and addressing the ‘VUS problem’ in genetic diagnosis of severe insulin resistance, this study, like the MC4R MAVE above, will help to stratify patients with newly identified INSR variants for potential targeted therapies with non-canonical INSR ligands including monoclonal antibodies.

### Other endocrine receptor classes and downstream signalling genes

Other important hormone receptors from different structural classes (e.g. cytokine-type receptors (exemplified by receptors for growth hormone and leptin) and TGF-family receptors (e.g. for GDF15) have yet to be reported. However, the experience of MAVEs in other receptor classes, as described above, offers a series of paradigms that may be adapted.

## Application of MAVEs to other endocrine disease-related genes

MAVE studies of endocrine disease-related genes beyond receptors remain limited. A major challenge, which applies broadly across MAVE applications, is that unlike receptors – where generalisable and well-established multiplexed assay platforms are available (for example, FACS antibodies to quantify surface expression or cAMP-responsive reporters to measure GPCR signalling) – non-receptor endocrine genes span diverse functional classes and require different assay systems. Nevertheless, examples of notable progress are seen for monogenic diabetes genes glucokinase (GCK), for which the effects of nearly all possible missense and nonsense variants on enzyme activity and protein abundance have been studied in yeast complementation-based MAVEs (114, 115), and HNF1A, for which deep mutational scanning has quantified the transcriptional effects of 11,970 protein-coding variants in human hepatocytes (116).

## Future opportunities and challenges in applying MAVEs to hormone receptors

The MAVE field is developing rapidly on many fronts, with continuous innovation not only in underpinning technology, its applications, and associated analytic pipelines, but also in its translational interface with genetic diagnostics and rare disease therapy. As methodologies become further streamlined, and as costs fall, endocrine MAVEs using a range of hormone concentrations, time courses, and multidimensional readouts are increasingly expected. Such multidimensional readouts may involve multiplexed reporters of different parts of complex receptor-activated signalling networks, single-cell transcriptomics, and in due course single-cell proteomics. Single-cell transcriptomics has particular appeal for nuclear hormone receptors, where different transcriptional responses may be differentially activated either by distinct ligands or in response to different mutational perturbations.

Another exciting possibility in future is the application of MAVEs to secreted peptide hormones. This may provide a route to novel peptide hormone analogues with a raft of desirable properties in different therapeutic situations, exemplified by the proliferation of insulin analogues in recent decades. Making a mutational library of a secreted peptide would be straightforward, so the challenge lies in retaining the coupling of expressed, secreted mutant peptides to the tagging barcode in the cell of origin. A step towards this was recently provided by a study that used fusion of secreted peptides retaining their own signal sequence to a transmembrane protein anchoring them to the plasma membrane (117). If this approach can be shown to generate cell surface-expressed peptide hormones with retained bioactivity, then a new array of exciting translational possibilities will open up. The MAVE train is gathering pace indeed, and the possibilities for translational endocrine research are great.

## Declaration of interest

RKS received consulting fees from AstraZeneca and Alnylam and speaking fees from Novo Nordisk, Eli Lilly, and Amryt, all on the subject of severe insulin resistance/lipodystrophy. The other authors declare no conflicts of interest.

## Funding

JX was funded by a studentship from Diabetes UK (23/0006652).

## Author contribution statement

JX and VA wrote the original draft of the manuscript. GK reviewed and edited the manuscript. RKS acquired funding and wrote, reviewed, and edited the original draft of the manuscript.
